# Risk Factors Associated With Lower Bone Mineral Density in Primary Aldosteronism Patients

**DOI:** 10.3389/fendo.2022.884302

**Published:** 2022-06-16

**Authors:** Xiaomei Lv, Huijun Hu, Chuyu Shen, Xiaoyun Zhang, Li Yan, Shaoling Zhang, Ying Guo

**Affiliations:** ^1^ Department of Endocrinology, Sun Yat-sen Memorial Hospital, Sun Yat-sen University, Guangzhou, China; ^2^ Department of Radiology, Sun Yat-sen Memorial Hospital, Sun Yat-sen University, Guangzhou, China

**Keywords:** primary aldosteronism, bone mineral density, quantitative CT, inflammation, oxidative stress, MPV, TBIL

## Abstract

**Purpose:**

The association between primary aldosteronism (PA) and lower bone mineral density (BMD) has raised a concern, but the contributing factors remain unclear. We aim to explore the risk factors for lower BMD in PA patients.

**Methods:**

We analyzed and compared the data of 60 PA patients with 60 matched essential hypertension (EH) patients. BMD, bone metabolites, and several oxidative stress and inflammation indicators—including C-reactive protein (CRP), superoxide dismutase (SOD), total bilirubin (TBIL), mean platelet volume (MPV), *etc.*—were assessed and compared in PA and EH patients. Bivariate correlation analysis and multivariate linear regression analysis were performed to explore the factors associated with BMD in PA patients.

**Results:**

The BMD measured by quantitative computed tomography in PA patients was lower than that in EH patients (141.9 ± 34.0 vs. 158.9 ± 55.9 g/cm^3^, *p* = 0.047), especially in patients less than 50 years old. BMD was independently negatively associated with age (standardized *β* = -0.581, *p* < 0.001), serum phosphorus (standardized *β* = -0.203, *p* = 0.008), urinary calcium excretion (standardized *β* = -0.185, *p* = 0.031), and MPV (standardized *β* = -0.172, *p* = 0.043) and positively associated with SOD (standardized *β* = 0.205, *p* = 0.011) and TBIL (standardized *β* = 0.212, *p* = 0.015).

**Conclusions:**

The PA patients showed a lower BMD than the EH patients, which was associated with age, serum phosphorus, urinary calcium excretion, MPV, SOD, and TBIL. These variables might be potential markers for the assessment of bone loss and efficacy of treatments in PA patients.

## Introduction

Primary aldosteronism (PA) is the most common endocrine-related hypertension, which frequently manifests as hypertension and hypokalemia (1, 2). Aldosterone overproduction was reported to be a risk factor leading to cardiovascular, renal, and metabolic diseases (3). Recent studies also found that PA was related to a higher risk of impaired bone mass, the most common presentations of which are lower bone mineral density (BMD) and higher risk of fractures (4–6). More and more convincing evidence suggested that PA might be a secondary cause of osteoporosis (7).

Osteoporosis is the most common bone disease characterized by low bone mass and deterioration of bone microstructure, resulting in increased bone brittleness and increased risk of fractures, which as decreases the quality of life of PA patients. Inflammation and oxidative stress were reported to contribute to the pathogenic mechanisms of osteoporosis, mainly through activating osteoclastogenesis and inhibiting osteoblastogenesis (8–10). Besides this, several indicators of inflammation and oxidative stress were increased in PA patients (11, 12), implicating an association between PA and inflammation and oxidative stress. However, the mechanism of association between PA and bone impairment remains unclear, and whether inflammation and oxidative stress were associated with impaired bone mass in PA patients has not been investigated yet.

On the basis of these premises, in this study, we aim to assess the prevalence of impaired bone mass in PA and EH patients and explore whether inflammation and oxidative stress have an impact on BMD in PA patients.

## Methods

### Study Population

**Figure 1:**
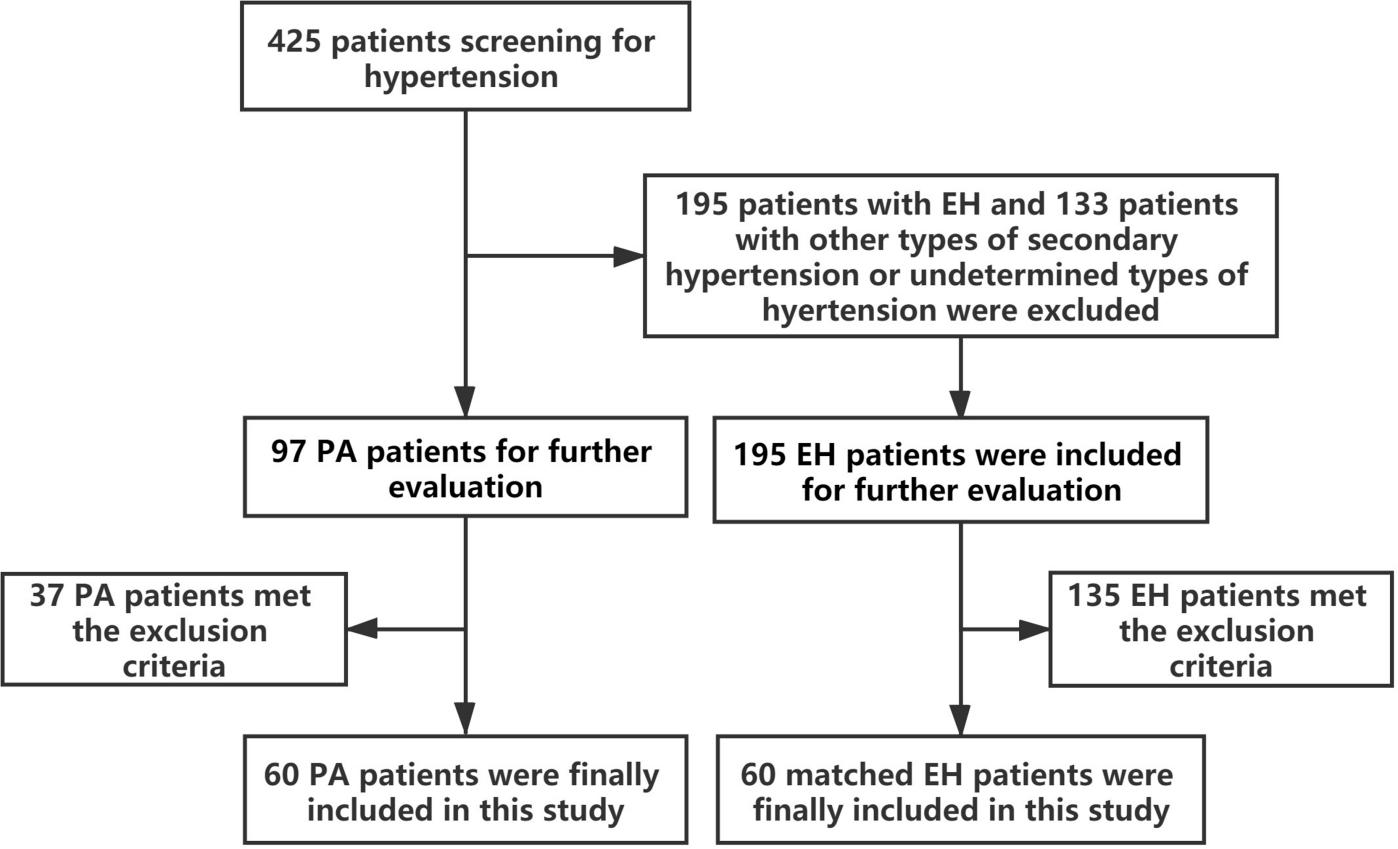
Participants’ flow chart of primary aldosteronism and essential hypertension patients in this study.

This was a single-center, case-controlled study carried out at the Department of Endocrinology, Sun Yat-sen Memorial Hospital of Sun Yat-sen University in Guangzhou, China. From January 2020 to June 2021, a total of 425 patients referred to our center for screening for cause of hypertension were enrolled in our study. After excluding patients who did not meet the recruitment requirements, the data of 60 PA patients and 60 EH patients matched by sex, age ( ± 2 years), body mass index (BMI, ± 2 kg/m^2^), duration of hypertension ( ± 2 years), and blood pressure (BP, ± 5 mmHg) were finally analyzed in our study (**Figure 1**). This study was reviewed and approved by the Ethics Committee of Sun Yat-sen Memorial Hospital. Informed consent has been obtained from the patients before enrollment.

### Case Definition

All enrolled patients obtained a comprehensive evaluation for screening for a secondary cause of hypertension according to clinical features. Briefly, examinations of aldosterone–renin ratio (ARR), serum and urine catecholamine metabolite concentrations, and serum and urine cortisol levels as well as 1-mg overnight dexamethasone suppression test and renal computed tomography angiography were performed routinely to screen for PA, PA with cortisol co-secretion (13, 14), pheochromocytoma, hypercortisolism, and renovascular hypertension. Other examinations were performed to screen for other secondary causes of hypertension as clinically indicated.

The newly confirmed PA patients were included in the PA group. The screening and case definition for PA were according to the PA guidelines (1, 2). Before screening for PA, all drugs that affect the result of ARR were required to be withdrawn to make the ARR convincing. In a nutshell, the diagnostic criteria of PA were defined as a positive screening test for PA, which, in our study, was followed by at least one positive confirmatory test, including captopril challenge test, saline infusion test, furosemide-upright test, and oral salt-loading test. A differential diagnosis of PA forms (bilateral PA and unilateral PA) was derived according to adrenal high-resolution computerized tomography and/or magnetic resonance imaging and by adrenal vein sampling (AVS). Specifically, unilateral PA was confirmed according to the findings of AVS and/or a combination of computerized tomography and/or magnetic resonance imaging finding of pathologically confirmed unilateral adrenocortical adenoma and/or normalization of plasma renin activity (PRA) and plasma aldosterone concentration (PAC) levels after laparoscopic adrenalectomy. The AVS procedure and criteria were according to published guidelines (15).

The EH control group included patients with a history of hypertension and who were receiving treatment such as antihypertensive drugs or with three office BP measurements of systolic blood pressure ≥140 mmHg and/or diastolic blood pressure (DBP) ≥90 mmHg at different days after excluding a secondary cause of hypertension with comprehensive evaluation.

The exclusion criteria in this study were as follows: (1) patients with a current history of known secondary causes of osteoporosis such as thyroid disorders, inflammatory bowel diseases, rheumatoid arthritis, chronic obstructive pulmonary disease, and primary hyperparathyroidism; (2) patients who were receiving a treatment that affects the results of BMD and bone turnover markers such as glucocorticoid treatment (≥5 mg prednisolone daily or equivalent for 3 months or more), diphosphonate, denosumab, vitamin D, calcium supplementation, and hormone replacement therapy; (3) patients who were pregnant or lactating women; (4) patients with a history of non-osteoporotic fracture within 6 months; (5) patients with incomplete medical records; (6) patients with other confirmed or suspected secondary causes of hypertension except for PA; and (7) PA patients who had been treated with mineralocorticoid receptor antagonists or had been subjected to adrenalectomy.

### Clinical Characteristics and Laboratory Measurements

Information on gender, age, BMI, BP, hypertension duration, smoking history, drinking history, and physical exercise were collected from medical records. BMI was calculated as weight (kg)/height (m)^2^. BP was defined as the average value of three office BP measurements after the patients had been seated quietly for at least 10 min at different days.

The laboratory parameters of serum potassium, calcium, phosphorus, creatinine, albumin, glycosylated hemoglobin (HbA1c), superoxide dismutase (SOD), total bilirubin (TBIL), uric acid, C-reactive protein (CRP), and urinary calcium and phosphorus concentration were measured by an automatic biochemical analyzer (AU5800, Beckman Coulter, USA). The calculation of estimated glomerular filtration rate was based on the CKD-EPI equation. Peripheral blood cell indices, including the count of white blood cells, lymphocytes, neutrophils, monocytes, platelets, and MCV were measured by an automatic analyzer (DxFLEX, Beckman Coulter, USA). Bone metabolites, including intact parathyroid hormone (iPTH) and calcitonin, were measured by using chemiluminescent immunoassay kits (Siemens, Gwynedd, UK), bone alkaline phosphatase (BLP) and 25-hydroxyvitamin D were measured by commercial ELISA kits (IDS, Boldon, UK), and N-terminal mid osteocalcin (N-MID-OT), type I procollagen N-terminal peptide (PINP), and β-Crosslaps were measured by chemiluminescent immunoassay kits (Roche, Mannheim, Germany). The measurements of PAC and PRA were determined by using chemiluminescent immunoassay kits (Snibe, Shenzhen, China). The reference range values of bone metabolites were as follows: iPTH (11.0–47.0 pg/ml), calcitonin (<2.0 pg/ml), BLP (3.0–15.0 ug/L), 25-hydroxyvitamin D (75.0–250.0 nmol/L), N-MID-OT (11.0–46.0 ng/ml), PINP (14.3–76.3 ng/ml), and β-Crosslaps (≤1.01 ng/ml). The reference range of PAC was 7.0–30.0 ng/dl and that of PRA was 0.10–6.56 ng/ml/h in the standing position.

### BMD Measurement

BMD was measured by quantitative computed tomography (QCT), an effective tool to measure the volumetric BMD of patients and which could measure the volume density of cancellous bone and cortical bone, respectively. Compared with dual-energy X-ray absorptiometry, BMD measured by QCT was not affected by spinal hyperplasia, degeneration, and vascular calcification. In this study, BMD was measured using QCT (Siemens, Germany) with QCT volume model calibration and professional software analysis at average at the lumbar spine (T12-L3), and at least two intact vertebrae were measured (16). According to the guidelines for the diagnosis criteria of osteoporosis with QCT (16, 17), normal bone mass was defined as BMD >120 g/cm^3^, and 80 g/cm^3^ ≤ BMD ≤120 g/cm^3^ was defined as osteopenia, whereas BMD <80g/cm^3^ was defined as osteoporosis. In this study, we divided the patients into the osteopenia group (BMD ≤120 g/cm^3^) and the normal bone mass group (BMD >120 g/cm^3^).

### Statistical Analysis

We used SPSS version 25.0 (SPSS, Inc., Chicago, IL, USA) for all statistical analysis. Normality test and analysis of variance were performed on all recorded data. Continuous variables in normal distribution were expressed by mean and standard deviation, and continuous variables in the skew distribution were expressed by median and interquartile range. Frequencies or percentages were expressed in categorical variables. Student’s *t*-test, Mann–Whitney *U*-test, or chi-square test were used to test the group differences between PA and EH patients when appropriate. Spearman or Pearson correlation analysis and partial correlation analysis were used to study the correlation between BMD and laboratory parameters. Multivariate linear regression analysis was performed to explore independent association between clinical factors and BMD in the PA group. Assumptions of multivariate linear regression, including linearity, normality, homoscedasticity, and collinearity, were tested and found to be satisfactory. *P <*0.05 in a two-tailed test was defined as statistical significance.

## Results

### Characteristics Between PA and EH Groups

The clinical and biochemical characteristics of PA and EH groups are shown in **Table 1**. We found that BMD in PA patients was lower than that in EH patients (141.9 ± 34.0 *vs*. 158.9 ± 55.9 g/cm^3^, *p* = 0.047), while the prevalence of osteopenia showed no significant difference. PA patients presented a higher urinary calcium concentration [6.0 (5.2–6.5) *vs*. 4.8 (3.4–4.9) mmol/L, *p <*0.001], higher iPTH [49.5 (39.5–62.5) *vs*. 36.0 (28.0–48.5) pg/ml, *p <*0.001], lower serum potassium (3.37 ± 0.34 *vs*. 3.90 ± 0.30 mmol/L, *p <*0.001), lower serum phosphorus (1.08 ± 0.14 *vs*. 1.14 ± 0.16 mmol/L, *p* = 0.042), and lower TBIL [11.7 (10.5–14.0) *vs*. 13.0 (11.7–15.9) μmol/L, *p* = 0.038] than EH patients.

**Table 1:** Comparison of characteristics between the PA and EH groups.

Variables	PA group (*n* = 60)	EH group (*n* = 60)	*P*-value
Age (years)	48.8 ± 13.1	48.4 ± 13.8	0.892
Male (*n*, %)	29/48.3%	29/48.3%	1.000
BMI (kg/m^2^)	24.7 ± 3.7	25.3 ± 3.8	0.360
Hypertension duration (months)	54 (18–120)	36 (12–99)	0.488
SBP (mmHg)	140.5 ± 13.8	142.0 ± 13.9	0.547
DBP (mmHg)	86.4 ± 10.2	86.9 ± 10.0	0.780
BMD (g/cm^3^)	141.9 ± 34.0	158.9 ± 55.9	0.047*
Osteopenia (*n*, %)	19/31.6%	18/30.0%	0.843
Smoking history (*n*, %)	15/24.6%	12/25.7%	0.499
Drinking history (*n*, %)	7/13.1%	12/21.2%	0.088
Physical exercise >30 min/day (*n*, %)	20/36.1%	15/24.8%	0.709
PAC (ng/dl)	31.0 (26.8–49.8)	21.8 (14.3–28.7)	<0.001*
PRA (ng/ml/h)	0.18 (0.07–0.63)	4.91 (1.59–8.33)	<0.001*
ARR (ng/dl per ng/ml per h)	171.4 (64.9–1,037.4)	4.4 (2.7–12.1)	<0.001*
HbA1c (%)	5.5 (5.2–5.7)	5.4 (5.1–5.8)	0.491
Serum potassium (mmol/L)	3.37 ± 0.34	3.90 ± 0.30	<0.001*
Serum calcium (mmol/L)	2.30 ± 0.10	2.33 ± 0.19	0.363
Serum phosphorus (mmol/L)	1.08 ± 0.14	1.14 ± 0.16	0.042*
24-h urinary calcium (mmol/L)	6.0 (5.2–6.5)	4.8 (3.4–4.9)	<0.001*
24-h urinary phosphorus (mmol/L)	13.4 (12.8–17.3)	15.2 (13.0–15.8)	0.141
iPTH (pg/ml)	49.5 (39.5–62.5)	36.0 (28.0–48.5)	<0.001*
Calcitonin (pg/ml)	2 (2–2)	2 (2–2)	0.916
BLP (ug/L)	17.0 (14.0–23.0)	19.5 (15.0–26.8)	0.168
25-Hydroxyvitamin D (nmol/L)	43.0 (39.0–59.6)	48.6 (41.2–64.3)	0.083
B-crosslaps (ng/ml)	0.5 (0.3–0.6)	0.4 (0.3–0.5)	0.109
N-MID-OT (ng/ml)	13.4 (10.9–19.6)	13.8 (10.1–16.6)	0.587
PINP (ng/ml)	46.3 (39.6–63.9)	51.6 (32.7–64.6)	0.979
SOD (U/ml)	167.4 ± 15.0	170.5 ± 16.2	0.276
Albumin (g/l)	40.1 ± 3.5	41.0 ± 3.8	0.176
TBIL (μmol/L)	11.7 (10.5–14.0)	13.0 (11.7–15.9)	0.038*
UA (μmol/L)	384.0 (282.5–445.8)	383.5 (288.3–434.0)	0.881
CRP (mg/L)	1.1 (0.6–1.9)	1.2 (0.5–1.7)	0.669
eGFR (ml/min per 1.73 m^2^)	91.5 ± 24.1	95.5 ± 22.7	0.269
WBC (*10^9^/L)	6.7 (5.1–8.2)	6.8 (5.6–7.9)	0.973
Lymphocytes (*10^9^/L)	1.7 (1.4–2.0)	1.7 (1.4–2.1)	0.944
Neutrophils (*10^9^/L)	4.0 (3.0–5.4)	4.4 (3.3–5.3)	0.773
Monocytes (*10^9^/L)	0.44 (0.36–0.63)	0.48 (0.37–0.57)	0.975
Platelet (*10^9^/L)	244.0 (211.3–285.5)	257.5 (210.3–313.3)	0.448
MPV (fl)	10.5 (9.6–11.1)	10.3 (9.7–10.7)	0.263
PA subtypes			
Unilateral PA (*n*, %)	35/58.3%	–	–
Bilateral PA (*n*, %)	25/41.7%	–	–

Data are shown as mean ± standard deviation or as median (25th–75th percentile) or as numbers or percentages.

*p <0.05 was defined as statistically significant.

ARR, aldosterone-renin ratio; BLP, bone alkaline phosphatase; BMD, bone mineral density; BMI, body mass index; CRP, C-reactive protein; DBP, diastolic blood pressure; eGFR, estimated glomerular filtration rate; EH, essential hypertension; HbA1c, glycosylation hemoglobin; MPV, mean platelet volume; N-MID-OT, N-terminal mid osteocalcin; PA, primary aldosteronism; PAC, plasma aldosterone concentration; PINP, type I procollagen N-terminal peptide; PRA, plasma renin activity; iPTH, intact parathyroid hormone; SBP, systolic blood pressure; SOD, superoxide dismutase; TBIL, total bilirubin; UA, uric acid; WBC, white blood cell count.

### Characteristics Between PA and EH Patients According to Age Stratification of 50 Years Old

The mean menopausal age in this study was 50 years old (**Supplementary Table S1**). After the stratification of menopause, the difference of BMD between PA and EH patients only existed in non-menopausal patients (**Supplementary Table S1**). After applying further stratification according to 50 years old in male patients, the significance for BMD between PA and EH patients was only present in the subgroup of age below 50 years old, while the other variables that presented a significant difference between PA and EH patients were similar to those of the female subgroups (**Supplementary Table S2**).

Thus, to minimize the influence of menopause (18), we further divided the patients into groups <50 years old and ≥50 years old. As shown in **Table 2**, the gender proportion and the prevalence of osteopenia showed no significant difference between PA and EH patients in both groups. In the group <50 years old, the PA patients presented a lower BMD (165.0 ± 21.0 *vs*. 199.7 ± 31.1 g/cm^3^, *p* < 0.001), lower serum potassium (3.36 ± 0.39 *vs*. 3.88 ± 0.32 mmol/L, *p* < 0.001), lower serum phosphorus (1.08 ± 0.13 *vs*. 1.17 ± 0.18 mmol/L, *p* = 0.026), higher urinary calcium excretion [6.0 (4.7–6.1) *vs*. 4.8 (4.0–5.0) mmol/L, *p* = 0.033], and higher iPTH [49.8 (40.5–68.5) *vs*. 36.5 (26.8–50.5) pg/ml, *p* = 0.003) than the EH control group.

**Table 2:** Comparison of characteristics between PA and EH patients according to age stratification of 50 years old.

Group	<50 years old			≥50 years old		
Variables	PA group (*n* = 30)	EH group (*n* = 30)	*P*-value	PA group (*n* = 30)	EH group (*n* = 30)	*P*-value
Age (years)	37.6 ± 7.0	36.7 ± 7.1	0.622	59.9 ± 6.8	60.1 ± 7.5	0.900
Male (*n*, %)	13/43.3%	13/43.3%	1.000	16/53.3%	16/53.3%	1.000
BMI (kg/m^2^)	24.3 ± 4.4	25.4 ± 4.4	0.312	25.0 ± 2.8	25.1 ± 3.2	0.901
Hypertension duration (months)	24.0 (6.0–54.0)	18.0 (4.0–42.0)	0.583	90.0 (54.0–180.0)	79.0 (16.0–157.5)	0.278
SBP (mmHg)	140.9 ± 13.4	144.7 ± 14.3	0.288	140.1 ± 14.4	139.4 ± 13.1	0.825
DBP (mmHg)	90.3 ± 9.0	92.3 ± 9.7	0.412	82.5 ± 9.9	81.5 ± 7.0	0.665
BMD (g/cm^3^)	165.0 ± 21.0	199.7 ± 31.1	<0.001*	119.7 ± 30.6	118.1 ± 44.1	0.867
Osteopenia (*n*, %)	1/1.7%	0/0%	–	18/60.0%	18/60.0%	1.000
PA subtypes						
Unilateral PA (*n*, %)	18/60.0%	–	–	17/56.7%	–	–
Bilateral PA (*n*, %)	12/40.0%	–	–	13/43.3%	–	–
PAC (ng/dl)	30.9 (27.6–46.3)	25.3 (14.0–30.1)	<0.001*	33.2 (24.1–50.0)	20.8 (14.0–30.1)	<0.001*
PRA (ng/ml/h)	0.09 (0.01–0.20)	6.29 (2.17–10.61)	<0.001*	0.28 (0.08–1.21)	3.21(1.04–6.21)	<0.001*
ARR (ng/dl per ng/ml per h)	360.3 (167.9–2608.3)	4.0 (2.5–7.9)	<0.001*	81.3 (49.1–433.8)	5.6 (3.0–15.0)	<0.001*
Serum potassium (mmol/L)	3.36 ± 0.39	3.88 ± 0.32	<0.001*	3.39 ± 0.28	3.92 ± 0.29	<0.001*
Serum calcium (mmol/L)	2.29 ± 0.08	2.32 ± 0.26	0.547	2.31 ± 0.11	2.34 ± 0.09	0.423
Serum phosphorus (mmol/L)	1.08 ± 0.13	1.17 ± 0.18	0.026*	1.09 ± 0.16	1.11 ± 0.14	0.564
24-h urinary calcium (mmol/L)	6.0 (4.7–6.1)	4.8 (4.0–5.0)	0.033*	6.0 (5.6–6.9)	4.8 (2.9–4.8)	<0.001*
24-h urinary phosphorus (mmol/L)	13.4 (13.2–17.6)	15.2 (14.8–18.1)	0.064	13.4 (12.2–16.0)	14.5 (11.2–15.2)	0.964
iPTH (pg/ml)	49.8 (40.5–68.5)	36.5 (26.8–50.5)	0.003*	49.5 (34.0–53.8)	36.0 (33.5–47.0)	0.019*
25-Hydroxyvitamin D (nmol/L)	39.9 (34.4–47.0)	48.2 (39.5–62.1)	0.092	44.7 (43.0–72.6)	48.6 (43.7–71.2)	0.468
SOD (U/ml)	170.0 ± 15.3	176.2 ± 15.2	0.120	164.8 ± 14.5	65.8 ± 15.2	0.998
TBIL (μmol/L)	11.7 (10.5–14.1)	13.0 (10.6–15.3)	0.321	11.7 (10.7–14.4)	13.0 (11.4–16.3)	0.044*
UA (μmol/L)	384.5 (290.5–447.8)	390.0 (327.0–474.0)	0.540	378.0 (274.3–449.8)	367.0 (268.0–413.0)	0.734
CRP (mg/L)	1.1 (0.8–1.9)	1.2 (0.5–2.2)	0.848	1.1 (0.6–3.0)	1.2 (0.4–1.5)	0.723
MPV (fl)	10.5 (9.6–10.7)	10.3 (9.5–10.7)	0.589	10.7 (9.6–11.5)	10.3 (9.7–10.9)	0.300

Data are shown as mean ± standard deviation or as median (25th–75th percentile) or as numbers or percentages.

*p <0.05 was defined as statistically significant.

ARR, aldosterone-renin ratio; BMD, bone mineral density; CRP, C-reactive protein; DBP, diastolic blood pressure; EH, essential hypertension; MPV, mean platelet volume; PA, primary aldosteronism; PAC, plasma aldosterone concentration; PRA, plasma renin activity; iPTH, intact parathyroid hormone; SBP, systolic blood pressure; SOD, superoxide dismutase; TBIL, total bilirubin; UA, uric acid.

However, in the group of ≥50 years old, no significant difference was found in BMD between PA and EH patients (**Table 2**). The PA patients merely showed a lower serum potassium (3.39 ± 0.28 *vs*. 3.92 ± 0.29 mmol/L, *p* < 0.001), lower TBIL [11.7 (10.7–14.4) *vs*. 13.0 (11.4–16.3) μmol/L, *p* = 0.044], higher urinary calcium concentration [6.0 (5.6–6.9) *vs*. 4.8 (2.9–4.8) mmol/L, *p* < 0.001], and higher iPTH [49.5 (34.0–53.8) *vs*. 36.0 (33.5–47.0) pg/ml, *p* = 0.019] than the EH patients.

### Correlation Between BMD and Clinical and Laboratory Indices

Correlation analyses between BMD and clinical and laboratory indices in PA and EH patients were performed. As presented in **Table 3**, in PA patients, BMD was negatively correlated with age (*r* = -0.688, *p* < 0.001), PRA (*r* = -0.379, *p* = 0.003), hypertension duration (*r* = -0.409, *p* = 0.001), HbA1c (*r* = -0.311, *p* = 0.016), serum phosphorus (*r* = -0.265, *p* = 0.041), 25-hydroxyvitamin D (*r* = -0.381, *p* = 0.003), MPV (*r* = -0.366, *p* = 0.004), and urinary calcium concentration (*r* = -0.418, *p* = 0.001) and positively correlated with ARR (*r* = 0.374, *p* = 0.003), lymphocyte count (*r* = 0.267, *p* = 0.039), monocyte count (*r* = 0.296, *p* = 0.021), SOD (*r* = 0.272, *p* = 0.035), and TBIL (*r* = 0.349, *p* = 0.006). Surprisingly, age was positively correlated with PRA and negatively correlated with ARR (**Supplementary Table S3**). After eliminating the effect of age, BMD showed no partial correlation with PRA or ARR in PA patients (**Supplementary Table S4**). In EH patients, BMD was negatively correlated with age (*r* = -0.787, *p* < 0.001), hypertension duration (*r* = -0.440, *p* < 0.001), and HbA1c (*r* = -0.258, *p* = 0.047) and positively associated with DBP (*r* = 0.554, *p* < 0.001) and SOD (*r* = 0.313, *p* = 0.015).

**Table 3:** Bivariate correlation analysis of variables associated with BMD.

Group	PA group (*n* = 60)		EH group (*n* = 60)	
Variables	*r*	*P*-value	*r*	*P*-value
Age (years)	-0.688	<0.001*	-0.787	<0.001*
PRA (ng/ml/h)	-0.379	0.003*	–	NS
ARR (ng/dl per ng/ml per h)	0.374	0.003*	–	NS
DBP (mmHg)	0.359	0.005	0.554	<0.001*
Hypertension duration (months)	-0.409	0.001*	-0.440	<0.001*
HbA1c (%)	-0.311	0.016*	-0.258	0.047
Serum phosphorus (mmol/L)	-0.265	0.041*	–	NS
24-h urinary calcium (mmol/L)	-0.418	0.001*	–	NS
25-Hydroxyvitamin D (nmol/L)	-0.381	0.003*	–	NS
Lymphocyte count (*10^9^/L)	0.267	0.039	–	NS
Monocyte count (*10^9^/L)	0.296	0.021*	–	NS
SOD (U/ml)	0.272	0.035*	0.313	0.015
TBIL (μmol/L)	0.349	0.006*	–	NS
MPV (fl)	-0.366	0.004*	–	NS

*p <0.05 was defined as statistically significant.

ARR, aldosterone-renin ratio; HbA1c, glycosylation hemoglobin; MPV, mean platelet volume; NS, non-significant; PRA, plasma renin activity; SOD, superoxide dismutase; TBIL, total bilirubin.

### Multivariate Linear Regression Analysis of Factors Associated With BMD in PA Patients

We further performed multivariate linear regression analysis to investigate the clinical factors associated with BMD in PA patients independently. Variables that showed a significant association with BMD in a bivariate correlation analysis were included in the multivariate regression analysis using stepwise regression. As presented in **Table 4**, we found that BMD was independently negatively associated with age (standardized *β* = -0.581 *p* < 0.001), serum phosphorus concentration (standardized *β* = -0.203, *p* = 0.008), urinary calcium excretion (standardized *β* = -0.185, *p* = 0.031), and MPV (standardized *β* = -0.172, *p* = 0.043) and positively associated with SOD (standardized *β* = 0.205, *p* = 0.011) and TBIL (standardized *β* = 0.212, *p* = 0.015).

**Table 4:** Multivariate linear regression analysis of factors associated with BMD in PA patients.

Variables	Unstandardized regression coefficients (*β*_1_)	Standardized regression coefficients (*β*_2_)	95% CI	*P*-value
Age (years)	-1.842	-0.581	-2.342–1.341	<0.001*
Serum phosphorus (mmol/L)	-56.356	-0.203	-97.481–15.231	0.008*
24h urinary calcium (mmol/L)	-3.915	-0.185	-7.462–0.369	0.031*
MPV (fl)	-7.185	-0.172	-14.119–0.252	0.043*
SOD (U/ml)	0.567	0.205	0.135–0.999	0.011*
TBIL(μmol/L)	2.164	0.212	0.432–3.897	0.015*

*p <0.05 was defined as statistically significant.CI, confidence interval; MPV, mean platelet volume; SOD, superoxide dismutase; TBIL, total bilirubin.

## Discussion

In our study, we found that BMD was lower in PA patients than in EH patients, especially in patients less than 50 years old. Moreover, this study implicated that BMD was associated with age, serum phosphorus, urinary calcium excretion, MPV, SOD, and TBIL levels independently in PA patients.

Decreasing BMD was widely known to be closely associated with aging (19, 20). It was reported that about 33% of women over age 50 years would experience osteoporotic fractures as would 20% of men aged over 50 years (21). Osteoporosis was more common in postmenopausal women, which was related to the effect of estrogen. The mean menopausal age in Chinese women was 47.5–49.5 years old (18). In this study, we not only found that BMD was negatively associated with age but also found that PA had more effect on BMD in patients less than 50 years old, which further supported the impact of PA on BMD. However, we could not find a significant difference of BMD between PA and EH patients over 50 years old. A potential explanation could be the impact of age on BMD. It was reported that the prevalence of osteopenia was more than 60% among people over 50 years old in China (22). With an increase of age, the BMD of Czech women and men decreased by 68 and 58%, respectively (23). In our multivariate linear regression analysis, age was the most influential variable that affects BMD. We inferred from the above-mentioned observations that the missing level of significance for BMD between EH and PA patients over 50 years old might be due to the influence of age far exceeding those of other factors, including excess aldosterone.

In the present study, we discovered that BMD was negatively associated with serum phosphorus and urinary calcium excretion, suggesting that BMD was related to calcium and phosphorus metabolism disorders in PA patients. Actually, the association between PA and bone metabolism had been proposed. Several studies had suggested that PA patients presented a lower BMD compared with non-PA controls (6, 24, 25), and these studies unanimously showed higher urinary calcium excretion and higher PTH in PA patients, which was consistent with our results. Besides this, urinary calcium excrement and PTH were reduced and BMD was increased after the initiation of treatments of adrenalectomy or minerolocorticoid antagonists in PA patients (6, 24, 26). The possible mechanism of the increased urinary calcium excretion in PA patients was that volume expansion decreased the reabsorption of calcium by proximal renal tubules, and the amount of calcium secreted by distal renal tubules exceeded the amount of reabsorption, thus resulting in decreased serum calcium (24–27). Higher calcium excretion would decrease serum calcium and then possibly came secondary hyperparathyroidism to stabilize the calcium concentration by promoting the activity of osteoclasts and promoting the absorption of calcium in the intestine, finally resulting in bone loss in PA patients.

Unexpectedly, serum phosphorus was negatively associated with BMD in PA patients, and the 25-hydroxyvitamin D level was positively associated with BMD in a bivariate correlation analysis. The relationship between PA and 25-hydroxyvitamin D levels was controversial in previous studies. A previous study found that the 25-hydroxyvitamin D levels were decreased in PA patients (25), while others found no associations between them (24, 28, 29). The 25-hydroxyvitamin D levels were positively associated with urinary calcium excretion in elderly men (30–32). However, whether higher urinary calcium excretion could induce increased 25-hydroxyvitamin D levels had not been fully discussed. Although a different diet, sunbathing, and sports habits might explain the contradictory association between BMD and 25-hydroxyvitamin D levels, the results of our study raised the possibility that a higher urinary calcium excretion might increase the synthesis of 25-hydroxyvitamin D to maintain calcium and phosphorus homeostasis by promoting the absorption of intestinal calcium and phosphorus in the early stage of PA; further investigations are required to support this opinion. We supposed that the association between serum phosphorus and BMD might be a reflection of the comprehensive effect of PTH and 25-hydroxyvitamin D.

However, although we found a lower BMD in PA patients, the prevalence of osteopenia showed no difference between the PA and EH groups. This study included a relatively small sample size of PA patients, and it was worth noticing that previous studies did not mention the duration of PA, which might be of great significance to BMD. The PA patients in our study were all newly diagnosed, so the bone impairment might be relatively mild.

This was the first study to show that increased MPV was associated with lower BMD in PA patients. The PA patients had a lower MPV than the normotensive subjects (33), which was similar to our results. MPV is a parameter of measurement of platelet size and an early indicator of platelet activation, which is found to be related to osteoporosis (34, 35). Platelet activation was a link between thrombosis and inflammation, and MPV was associated with low-grade inflammation (36) and might be used as a marker to reflect the severity of inflammation (37). PA patients presented increased inflammation markers, such as higher levels of intercellular adhesion molecule-1, interleukin-6, and tumor necrosis factor-alpha messenger RNA and protein (11, 38). It was widely accepted that chronic inflammation took part in the pathophysiological mechanisms of osteoporosis (8, 9). Pro-inflammatory cytokines such as interleukin-1, 6 and tumor necrosis factor-alpha were able to activate osteoclastogenesis and stimulate osteoclastic bone resorption through activating the receptor activator of nuclear factor-kappaB ligand (RANKL) (9). As a result, MPV might be used as a simple marker to reflect the inflammation status in PA patients and connect PA with osteoporosis.

Besides this, we discovered that the SOD and TBIL levels were positively associated with BMD in PA patients, implicating that oxidative stress might have an effect on BMD in PA patients. As we know, SOD was an important regulator in oxidative stress, which catalyzed superoxides to convert into oxygen and hydrogen peroxides to reduce the levels of reactive oxygen species (39). TBIL could be considered as an antioxidant and anti-inflammatory, and the antioxidant capacity was even stronger than that of alpha-tocopherol, SOD, and catalase (40, 41). PA was associated with increased levels of oxidative stress indicators, including plasma NADPH oxidase, oxidized low-density lipoprotein, 8-isoprostane, *etc.*, and lower levels of superoxides and lipid peroxides were observed after spironolactone treatment (12, 42). Oxidative stress played an essential role in bone remodeling. Increased oxidative stress would induce bone loss by increasing RANKL expression and decreasing the differentiation and activities of osteoblasts through regulating kinases and transcription factor activities, including activating c-Jun N terminal kinases, Wnt/β-catenin, nuclear factor-kappaB signaling pathways, *etc. (* 10). Therefore, we considered that the SOD and TBIL levels could act as important indicators of oxidative stress status in PA patients and linked PA to a lower BMD in this study.

It was with regret that we could not find a direct correlation between BMD and the renin–angiotensin–aldosterone system (RAAS) components in our study. We speculated that the small sample size might be one of the reasons. In previous studies, RAAS activation was considered as a potential risk factor for osteoporosis. RAAS and mineralocorticoid receptors had been found to be expressed in human bone tissues, and increased angiotensin II and aldosterone might lead to increased bone turnover and decreased BMD (43, 44). In non-PA patients, it was reported that renin activity was positively and PAC was negatively associated with BMD (45, 46), while similar results were not be found in PA patients. However, the abnormality of RAAS in PA patients was featured by excess aldosterone and a suppressed renin–angiotensin system. An alternative reason for the missing correlation between BMD and RAAS components in PA patients might be the different effects of RAAS components to bone metabolism. As discussed above, potential mechanisms such as hyperparathyroidism, oxidative stress, and inflammation induced by excess aldosterone might play important roles in bone loss in PA patients, while the direct role of excess aldosterone and suppressed renin–angiotensin system in bone metabolism remained to be further investigated.

However, several limitations should be noted in our study. Firstly, this is a retrospective case–control study, so we cannot establish a causal relationship between PA and the lower BMD. Secondly, our study included a relatively small number of patients. Thirdly, we did not collect data on diet or sunbathing habit, which were important to bone metabolism. Therefore, studies that are better designed and with a larger sample size are needed for further investigations in PA and bone metabolism.

## Conclusion

In conclusion, PA patients showed a lower BMD than EH patients, especially in patients less than 50 years old. BMD was associated with age, serum phosphorus, urinary calcium excretion, MPV, SOD, and TBIL levels in PA patients, and these might be potential indicators of assessment of bone loss and the effect of treatments. We suggested that early attention should be paid to bone health problems in the PA population, and BMD measurement was recommended in the follow-up to improve the quality of life of the PA population.

## Data Availability Statement

The raw data supporting the conclusions of this article will be made available by the authors without undue reservation.

## Ethics Statement

The studies involving human participants were reviewed and approved by the Ethics Committee of Sun Yat-sen Memorial Hospital. The patients/participants provided their written informed consent to participate in this study.

## Author Contributions

XL, HH, and YG conceived and designed the study. XL, CS, HH, and XZ collected and managed the data. XL analyzed the data and wrote the manuscript. YG, HH, LY, and SZ reviewed and edited the manuscript. YG had a primary responsibility for final content. All authors contributed to the article and approved the submitted version.

## Funding

This work was supported by the Natural Science Foundation of Guangdong Province (grant number 2018A030313596) to YG.

## Conflict of Interest

The authors declare that the research was conducted in the absence of any commercial or financial relationships that could be construed as a potential conflict of interest.

## Publisher’s Note

All claims expressed in this article are solely those of the authors and do not necessarily represent those of their affiliated organizations, or those of the publisher, the editors and the reviewers. Any product that may be evaluated in this article, or claim that may be made by its manufacturer, is not guaranteed or endorsed by the publisher.

## Abbreviations

ARR, aldosterone-renin ratio; AVS, adrenal vein sampling; BLP, bone alkaline phosphatase; BP, blood pressure; BMD, bone mineral density; BMI, body mass index; CRP, C-reactive protein; DBP, diastolic blood pressure; eGFR, estimated glomerular filtration rate; EH, essential hypertension; HbA1c, glycosylation hemoglobin; MPV, mean platelet volume; N-MID-OT, N-terminal mid osteocalcin; PA, primary aldosteronism; PAC, plasma aldosterone concentration; PINP, type I procollagen N-terminal peptide; PRA, plasma renin activity; PTH, parathyroid hormone; QCT, quantitative computed tomography; RAAS, renin-angiotensin-aldosterone system; RANKL, receptor activator of nuclear factor-kappaB ligand; SBP, systolic blood pressure; SOD, superoxide dismutase; TBIL, total bilirubin; UA, uric acid; WBC, white blood cell count.
