# Supplementary material for: Risk Factors Associated With Lower Bone Mineral Density in Primary Aldosteronism Patients
Source: Front Endocrinol (Lausanne). 2022 Jun 16;13:884302. doi: 10.3389/fendo.2022.884302 (PMC9245341; doi:10.3389/fendo.2022.884302)
Supplement: Supplementary file 1 [file DataSheet_1.docx]

**Supplementary Table 1**

| **Supplementary Table 1 Characteristics of PA and EH patients stratified by menopausal status** | | | | | | |
| --- | --- | --- | --- | --- | --- | --- |
| group | Menopause status | | | Non-menopausal status | | |
| variables | PA group(n=14) | EH group(n=12) | P value | PA group(n=17) | EH group(n=19) | P value |
| Age (years) | 58.7±8.4 | 62.2±6.7 | 0.267 | 39.1±7.9 | 38.2±7.8 | 0.848 |
| Mean memopause (years old) | 50 | 50 | - | - | - | - |
| BMI (kg/m^2^) | 23.7±3.1 | 24.2±2.4 | 0.633 | 22.7±3.5 | 24.0±3.8 | 0.303 |
| Hypertension duration (months) | 75.0 (36.0-180.0) | 105.0 (18.0-18.0) | 0.856 | 24.0 (2.0-72.0) | 18.0 (6.0-36.0) | 0.937 |
| SBP (mmHg) | 135.3±16.9 | 135.4±12.6 | 0.979 | 142.6±11.8 | 141.4±13.9 | 0.777 |
| DBP (mmHg) | 81.1±10.0 | 80.0±6.5 | 0.526 | 90.5±9.1 | 90.2±11.2 | 0.926 |
| BMD (g/cm^3^) | 112.6±29.6 | 93.8±33.3 | 0.140 | 161.6±20.6 | 207.9±40.2 | **<0.001*** |
| Osteopenia (n, %) | 9/64.3% | 11/91.6% | 0.170 | 1/5.9% | 1/5.3% | 1.000 |
| PA subtypes |  |  |  |  |  |  |
| Unilateral PA (n,%) | 7/50.0% | - | - | 14/82.3% | - | - |
| Bilateral PA (n,%) | 7/50.0% | - | - | 3/17.6% | - | - |
| PAC (ng/dL) | 33.7 (18.5-49.4) | 19.8 (13.0-23.0) | **0.014*** | 30.9 (26.2-65.2) | 25.1 (14.1-28.9) | **0.002*** |
| PRA (ng/mL/hr) | 0.53 (0.12-2.46) | 4.07 (2.09-8.72) | **0.002*** | 0.11 (0.01-0.19) | 5.66 (1.95-10.42) | **<0.001*** |
| ARR (ng/dL per ng/mL per h) | 65.9 (40.7-272.1) | 3.1 (2.4-9.4) | **<0.001*** | 330.4 (164.3-2924.0) | 4.0 (2.5-12.3) | **<0.001*** |
| Serum potassium (mmol/L) | 3.30±0.39 | 3.91±0.37 | **<0.001*** | 3.37±0.36 | 3.81±0.31 | **<0.001*** |
| Serum calcium (mmol/L) | 2.33±0.10 | 2.35±0.08 | 0.759 | 2.28±0.09 | 2.33±0.10 | 0.159 |
| Serum phosphorus (mmol/L) | 1.18±0.16 | 1.13±0.09 | 0.348 | 1.10±0.11 | 1.15±0.18 | 0.295 |
| 24h urinary calcium (mmol/L) | 6.0 (5.4-7.3) | 4.7 (3.1-4.9) | **0.002*** | 6.0 (4.4-6.5) | 4.8 (3.9-6.1) | **0.010*** |
| 24h urinary phosphorus (mmol/L) | 13.4 (9.8-18.3) | 11.6 (9.1-14.8) | 0.381 | 13.4 (10.9-15.5) | 15.2 (13.8-15.4) | **0.037*** |
| iPTH (pg/mL) | 49.5 (39.3-66.8) | 33.0 (23.8-45.5) | **0.018*** | 53.0 (40.0-74.0) | 47.0 (32.0-60.0) | 0.163 |
| 25-hydroxyvitamin D (nmol/L) | 43.1 (42.0-50.3) | 54.5 (44.0-74.3) | 0.072 | 40.1 (36.2-44.3) | 49.4 (40.7-61.3) | **0.031*** |
| SOD (U/mL) | 164.1±15.4 | 164.5±18.7 | 0.945 | 169.4±15.3 | 174.0±13.0 | 0.338 |
| TBIL (μmol/L) | 11.1 (9.1-11.9) | 13.0 (10.4-15.5) | 0.135 | 11.7 (10.4-13.7) | 13.0 (9.3-14.5) | 0.557 |
| UA (μmol/L) | 331.5 (246.8-439.3) | 292.0 (218.8-404.4) | 0.504 | 354.0 (287.5-414.0) | 374.0 (285.0-411.0) | 0.824 |
| CRP (mg/L) | 0.9 (0.6-1.6） | 1.3 (0.6-1.5） | 0.589 | 1.1 (0.5-1.9) | 1.2 (0.3-2.2) | 0.886 |
| MPV (fL) | 10.7 (9.8-11.2) | 10.5 (9.9-10.9) | 0.503 | 10.2 (9.5-10.7) | 10.3 (9.7-10.7) | 0.787 |

Supplementary Table 1: Characteristics between PA and EH patients according to menopausal status.

Data were shown as mean ± standard deviation or as median (25^th^-75^th^ percentile) or as numbers or percentages. *p<0.05 was defined as statistical significance.

Abbreviations: ARR, aldosterone-renin ratio; BMD, bone mass density; CRP, C-reactive protein; DBP, diastolic blood pressure; EH, essential hypertension; MPV, mean platelet volume; PA, primary aldosteronism; PAC, plasma aldosterone concentration; PRA, plasma renin activity; iPTH, intact parathyroid hormone; SBP, systolic blood pressure; SOD, superoxide dismutase; TBIL, total bilirubin; UA, uric acid.

**Supplementary Table 2**

| **Supplementary Table 2 Characteristics of male PA and EH patients stratified by 50 years old** | | | | | | |
| --- | --- | --- | --- | --- | --- | --- |
| group | <50 years old | | | ≥50 years old | | |
| variables | PA group(n=13) | EH group(n=13) | P value | PA group(n=16) | EH group(n=16) | P value |
| Age (years) | 37.1±7.1 | 36.9±8.1 | 0.939 | 59.9±7.6 | 59.5±7.9 | 0.874 |
| BMI (kg/m^2^) | 26.9±4.4 | 27.9±4.0 | 0.531 | 25.8±2.2 | 25.5±3.7 | 0.780 |
| Hypertension duration (months) | 18.0 (6.5-51.0) | 12.0 (4.5-42.0) | 0.717 | 120.0 (63.0-180.0) | 66.0 (27.0-126.0) | 0.299 |
| SBP (mmHg) | 140.4±13.9 | 148.6±14.6 | 0.153 | 143.0±12.7 | 142.5±12.9 | 0.906 |
| DBP (mmHg) | 89.6±9.3 | 92.7±9.6 | 0.421 | 84.0±10.3 | 84.3±6.3 | 0.918 |
| BMD (g/cm^3^) | 165.8±25.7 | 183.6±14.3 | **0.039*** | 128.9±32.5 | 129.5±38.7 | 0.959 |
| Osteopenia (n, %) | 2/15.4% | 0/0.0% | - | 8/50.0% | 6/37.5% | 0.476 |
| PA subtypes |  |  |  |  |  |  |
| Unilateral PA (n,%) | 7/50.0% | - | - | 8/82.3% | - | - |
| Bilateral PA (n,%) | 6/50.0% | - | - | 8/17.6% | - | - |
| PAC (ng/dL) | 30.9 (28.0-42.9) | 25.5 (15.1-31.3) | **0.029*** | 37.8 (29.6-52.8) | 19.5 (12.7-27.3) | **<0.001*** |
| PRA (ng/mL/hr) | 0.09 (0.05-0.46) | 6.61 (3.70-9.89) | **<0.001*** | 0.23 (0.07-0.80) | 1.77 (0.58-5.77) | **0.002*** |
| ARR (ng/dL per ng/mL per h) | 334.4 (58.6-1506.5) | 4.0 (2.1-6.4) | **<0.001*** | 130.8 (41.5-956.2) | 11.1 (3.9-36.4) | **<0.001*** |
| Serum potassium (mmol/L) | 3.46±0.38 | 4.03±0.31 | **<0.001*** | 3.36±0.24 | 3.88±0.22 | **<0.001*** |
| Serum calcium (mmol/L) | 2.32±0.08 | 2.41±0.09 | **0.014*** | 2.29±0.11 | 2.32±0.10 | 0.394 |
| Serum phosphorus (mmol/L) | 1.05±0.15 | 1.19±0.16 | **0.022*** | 1.03±0.15 | 1.09±0.16 | 0.307 |
| 24h urinary calcium (mmol/L) | 5.7 (4.8-7.1) | 4.8 (4.2-4.8) | **0.029*** | 6.0 (4.9-7.1) | 4.7 (2.1-4.8) | **0.001*** |
| 24h urinary phosphorus (mmol/L) | 16.9 (13.4-18.7) | 15.2 (14.1-24.6) | 0.244 | 13.4 (12.1-16.9) | 15.2 (11.8-19.2) | 0.520 |
| iPTH (pg/mL) | 48.0 (32.0-51.3) | 26.0 (20.0-37.0) | **0.021*** | 49.5 (45.5-66.5) | 37.0 (36.0-45.5) | **0.023*** |
| 25-hydroxyvitamin D (nmol/L) | 41.0 (33.7-69.8) | 47.1 (36.9-66.1) | 0.626 | 52.5 (43.0-83.5) | 47.1 (42.5-60.3) | 0.497 |
| SOD (U/mL) | 174.7±14.7 | 178.9±17.1 | 0.513 | 162.1±13.0 | 164.0±13.6 | 0.679 |
| TBIL (μmol/L) | 11.7 (10.2-14.6) | 13.0 (12.0-17.8) | 0.182 | 11.7 (10.6-15.8) | 12.9 (11.7-16.6) | 0.534 |
| UA (μmol/L) | 395.0 (330.0-482.0) | 443.0 (374.5-536.0) | 0.249 | 391.5 (304.5-476.0) | 382.0 (316.0-413.5) | 0.624 |
| CRP (mg/L) | 1.3 (1.0-1.7） | 1.2 (0.7-1.8） | 0.555 | 1.2 (0.3-3.7) | 0.9 (0.3-1.6) | 0.346 |
| MPV (fL) | 10.6 (9.9-11.1) | 9.9 (9.5-10.7) | 0.199 | 10.3 (9.6-11.6) | 10.0 (9.6-10.9) | 0.664 |

Supplementary Table 2: Characteristics between male PA and EH patients according to age stratification of 50 years old.

Data were shown as mean ± standard deviation or as median (25^th^-75^th^ percentile) or as numbers or percentages. *p<0.05 was defined as statistical significance.

Abbreviations: ARR, aldosterone-renin ratio; BMD, bone mass density; CRP, C-reactive protein; DBP, diastolic blood pressure; EH, essential hypertension; MPV, mean platelet volume; PA, primary aldosteronism; PAC, plasma aldosterone concentration; PRA, plasma renin activity; iPTH, intact parathyroid hormone; SBP, systolic blood pressure; SOD, superoxide dismutase; TBIL, total bilirubin; UA, uric acid.

**Supplementary Table 3**

| **Supplementary Table 3 Bivariate correlation analysis among BMD, age and PRA, ARR.** | | | | | | | | |
| --- | --- | --- | --- | --- | --- | --- | --- | --- |
| variables | Age (years) | | BMD (g/cm^3^) | | PRA (ng/mL/hr) | | ARR (ng/dL per ng/mL per h) | |
|  | r | P value | r | P value | r | P value | r | P value |
| Age (years) | - | - | -0.688 | <0.001* | 0.362 | 0.005* | -0.378 | 0.003* |
| BMD (g/cm^3^) | -0.688 | <0.001* | - | - | -0.379 | 0.003* | 0.374 | 0.003* |
| PRA (ng/mL/hr) | 0.362 | 0.005* | -0.379 | 0.003* | - | - | -0.965 | <0.001* |
| ARR (ng/dL per ng/mL per h) | -0.378 | 0.003* | 0.374 | 0.003* | -0.965 | <0.001* | - | - |

Supplementary Table 3: Bivariate correlation analysis among BMD, age and PRA, ARR.

*p<0.05 was defined as statistical significance.

Abbreviations: ARR, aldosterone-renin ratio; BMD, bone mineral density; PRA, plasma renin activity.

**Supplementary Table4**

| **Supplementary Table** **4 Partial correlation analysis between BMD and PRA, ARR with age control** | | | | | |
| --- | --- | --- | --- | --- | --- |
| Variable control | | | BMD (g/cm^3^) | PRA (ng/mL/hr) | ARR (ng/dL per ng/mL per h) |
| Age (years) | BMD (g/cm3) | r | 1.000 | -0.148 | 0.132 |
|  |  | P value | - | 0.263 | 0.320 |
|  | PRA (ng/mL/hr) | r | -0.148 | 1.000 | -0.222 |
|  |  | P value | 0.263 | - | 0.091 |
|  | ARR (ng/dL per ng/mL per h) | r | 0.132 | -0.222 | 1.000 |
|  |  | P value | 0.320 | 0.091 | - |

Supplementary Table 4: Partial correlation analysis between BMD and PRA, ARR with age control.

Abbreviations: ARR, aldosterone-renin ratio; BMD, bone mineral density; PRA, plasma renin activity.
